# Transgenderism: Facts and fictions

**DOI:** 10.4103/0019-5545.44917

**Published:** 2009

**Authors:** Somasundaram O

**Affiliations:** Thanigai Illam, 23rd Cross, 30 Besant Nagar, Chennai, India

**Keywords:** Transgender, history, ancient India

## Abstract

The nosology associated with transgender phenomena is undergoing rapid revisions. This phenomenon is mentioned in ancient cultures and the allusions to it are variously described in the Indian literatures. The trials and tribulations of the isolated segment of the human population are surmised from two autobiographical accounts of writers. The measures to improve the life of the transgender population are suggested.

Some aspects of human sexuality have come to focus in recent times. Nosologies of sexual behavior are also of recent origin. Magnus Hirschfeld (1868-1935) the famous German Sexologist has coined the words transvestites and transsexuals in the beginning of the 20^th^ Century. He established Institute for Sexual Science in 1919 at Berlin, which was destroyed by Nazis in 1933 (It is interesting to recall that he himself was a homosexual).

Virginia Prince[1] coined the word transgenderism which is a blanket term for both transsexualism and transvestism and authored books like *Understanding cross dressing* and *seventy years in the trenches of the Gender wars*.

Another pioneer in the study of transgenderism was Harry Benjamin. He treated hundreds of patient and introduced Sex reassignment surgery at The Johns Hopkins Hospital. International Gender Dysphoria Association was named after him.

In the metamorphosis of gender identity disorder, the course of last four to five decades is detailed by Green R.[2] ICD-10[3] has separate entry for transsexualism (F64.0) and Gender identity disorder of children (F64.2). There are two entries for transvestism - Dual role transvestism (F64.1), Fetishistic transvestism (F65.1).

ICD-IV has excluded transsexualism as separate entity and includes it in the Gender identity disorder;[4] Children (302.6) Adolescents and Adults (302.85). There is only an entry for Transvestic Fetishism (302.3). There is an entry for Dual-role tranvestism in I.C.D-10 (f64.1). Bland[5] mentions two types of transvestism - transsexual and homosexual.

It is a well-known fact that the words denoting transgenderites have been in use in various ancient languages throughout the world. In Tamil, ancient grammar extant today is *Tolkappiam* (Literally the ancient literature c.2 century A.D). It refers to the phenomena as ‘*Pedu*’. Various ancient poets and religious leaders also refer to these phenomena in words like ‘*Ali*’ and ‘*Pedi*’.

It is highly interesting to note cross-gender and cross-gender behavior in the epics of Mahabharata and Ramayana.

## SIKANDINI - SIKANDI LEGEND

Bhishma won the three maids in *Swayamvara* on behalf of his step-brother. The eldest daughter Amba has already chosen Salva, the King Saubala mentally. But Bhishma and Salva rejected to marry her outright. To avenge Bhishma she performed various penances and was born as Sikandini to king Drupata. In her female personage, she served an ascetic, who blessed her to be converted to a male warrior, Sikandi. In the battle fought at Kurushetra, Arjuna fought Bhishma screened by Sikandi. Bhishma knowing his antecedent as a woman refused to take weapons against him and allowed Arjuna to defeat him.

## ARJUNA CROSS-GENDER AND CROSS-DRESSING AS BIRHANNALA

The Pandavas had to spend their last year of their exile *incognito*. This period was spent in the kingdom of Virata. Rajaji describes this scenario aptly. Arjuna replied: “I shall hide myself in the guise of a eunuch and serve the ladies of the court. I shall hide under a jacket the scars on my arms made by the constant chafing of the bow-string. When I rejected Urvasi's amorous overtures on the ground that she was like a mother unto me, she cursed me with loss of manhood: but through Indra's grace the curse would hold good only for a year and the time would be mine to choose. I shall serve out that year of loss of manhood now. Wearing bangles made of white conches, braiding my hair like a woman and clothing myself in female attire, I shall engage myself in menial work in the inner apartment of Virata's queen. I shall teach the women singing and dancing. And I shall seek service saying that I used to serve Draupadi in Yudhistra's court.”

Arjuna in the gesture of Birhannala fought with Kaurava on behalf of Prince Uttara. “Arriving in front of the Kauravas, he got down, prayed to God, removed the conch bangles from his hands, and put on leather gauntlets. He then tied a cloth on his flowing hair, stood facing the east, meditated on his armor, got into the chariot and gloried in the familiar feel of his famous Gandiva bow. In the ensuing battle, he defeated Kauravas.

Rama assures eunuchs of better treatment in his next *Avatar*. When Rama went to forest to fulfill the wishes of his dying father, the people of Ayotya followed him to the banks of Sarayu touched by their affection Rama requested the men and the women to go back. After fourteen years, when he came back to Ayotya he saw the mounts of sands in the riverbanks. Non-plussed he disbanded the mounts and saw the human skeleton, with glittering eyeballs. When he rejuvenated the skeleton, he saw the various eunuchs who had come with the Ayotya citizens. Rama realized his mistake in asking only the men and women to return home and he had forgotten to mention about the eunuchs.

He assured them that in his next *Avatar* as Lord Krishna the eunuchs would be born as singers to sing the praise of the Lord. Thus, the practice of eunuchs singing for the health of the newborn babies came into vogue.

*Aravan*[6] is the patron saint of the transsexuals of Tamilnadu. *Aravan* (Literally the son of snake) is the son of Arjuna by the Serpent Princess Ulupi. To get victory in Kurushetra war *Aravan* has to be offered as the human sacrifice. He makes a last request that he should be married and should enjoy the connubial bliss before his sacrifice. Krishna transforms himself as a beautiful virgin (Mohini) and offers herself to *Aravan*. Next day *Aravan* is sacrificed. Mohini adopts the widowhood.

From this time onwards *Aravan* becomes the patron saint of transsexuals of Tamilnadu. He is worshipped in the Koovagam temple in Villupuram district. Transsexuals all over the country assemble here on the *Chitrapournami* Day. The Mahabharata scene is enacted and the transsexuals adopt the widowhood in the temple.[7]

There are local legends about the transsexuals in Ajmir, Mewar, and Hyderabad. Mewar legend is associated with the patron saint *Potharaju Matha*. Legends in the various parts of the world about the phenomena are discussed by Green.[2]

Recently two of the transsexuals (who are known as *Aravanigal* in Tamil, literally the spouses of the son of the serpent) have written books about themselves. Their social and communal living and the various problems and discriminations, stigma etc., are well brought out.[8]

The castration and emasculanizing operations are done in extremely unhygienic conditions and by non-medical operators. It is needless to mention about morbidity and mortality. The wounds are covered with herbs and allowed to heal by themselves. There is no question of pain relief. There are no legal sanctions for this operation.

Appropriate steps should be taken to legalize the operations and they should be done by qualified experts under ideal conditions. The practice followed by sex reassignment surgical clinics in the West should be emulated.

The prevalence and the incidents of sexually transmitted diseases in these people are rife. Many of the State Governments are actively involved in the recognition of HIV/AIDS in these groups and educating them. It is unfortunately true that many of the transsexuals are active sex workers and both categories should not be equated. Sex education is a high priority in these high-risk populations.

The livelihood for this category is marginal. Most of them go out to the shops in the bazaars of the major towns and demand alms. The shopkeepers out of fear of displeasing these people and to do well in the business offer handsome donations. The transsexuals also take part in the various functions both auspicious and inauspicious of the major communities. Many of them are also good cooks. A sizeable portion is forced to engage in prostitution. A part of the income is spent for the castration operation. Many of them have got artistic talent and take part in many cultural activities, folklore, street plays. It is needless to say alcohol, substance misuse are rampant at this times.

## CONCLUSION

The American Psychiatric Association has de-pathologized homosexual behavior in 1973 and considers it to be normal-variant behavior. It is debatable whether these intellectual manoeuvre has solved the problems of the homosexual. They continue to have psychosexual, psychosocial, legal, cultural and religious problems and are still marginalized and stigmatized. If the same yardstick is applied to gender identity disorders, will we be solving the many problems faced by them? The minimum progress we can make with regard to our transgender segments is specializing in gender disorders and the associated problems of sex reassignment surgery under ideal conditions. The various physical, sexual, psychological, social, and legal problems have to be faced. It is gratifying to note that Tamilnadu State Government has established a panel under the social welfare department to look after the aforesaid problems. Old age pensions have been grant to some of the senior *aravanies*. granting ration cards, voter identity cards and voting rights are on the anvil.
